# Editorial: Adaptive immunity in local tissues

**DOI:** 10.3389/fimmu.2023.1200663

**Published:** 2023-05-01

**Authors:** Wenjie Zhang, Xuefeng Wang, Xiao He, Yuekang Xu

**Affiliations:** ^1^ Anhui Provincial Key Laboratory for Conservation and Exploitation of Biological Resources, School of Life Sciences, Anhui Normal University, Wuhu, China; ^2^ Department of Biochemistry and Molecular Biology, School of Biology and Basic Medical Sciences, Soochow University, Suzhou, China; ^3^ Department of Pathology, The University of Utah, Salt Lake City, UT, United States

**Keywords:** tissue immunology, adaptive immunity, T cells, B cells, immune regulation

Traditionally, it has been thought that adaptive immune cells are activated in secondary lymphoid organs and migrate to peripheral tissues to perform their effect functions. However, more and more evidence has demonstrated that there are also naïve or resident adaptive immune cells in peripheral tissues (1, 2), and tissue immunology is beginning to reveal how these resident adaptive immune cells are integrated with organ physiology during both tissue development and disease evolution. Due to their unique microenvironment, with its own composite of cytokines and metabolites, immune cells in these peripheral tissues might have developed special immune characteristics that are different from those of immune cells in secondary immune organs, and participate directly in the pathophysiological activities of the local tissues (**Figure 1**). Since peripheral tissues may well be the direct site of infection or inflammation, the behavior of the adaptive immune cells in the local tissues is more closely related to the development of diseases than that in systematic lymphoid organs, especially at early stages of the pathogenesis (3). Therefore, understanding the characteristics of adaptive immunity in different peripheral tissues may be useful to identify novel therapeutic targets to develop immediate and effective remedies for regional diseases.

**Figure 1:**
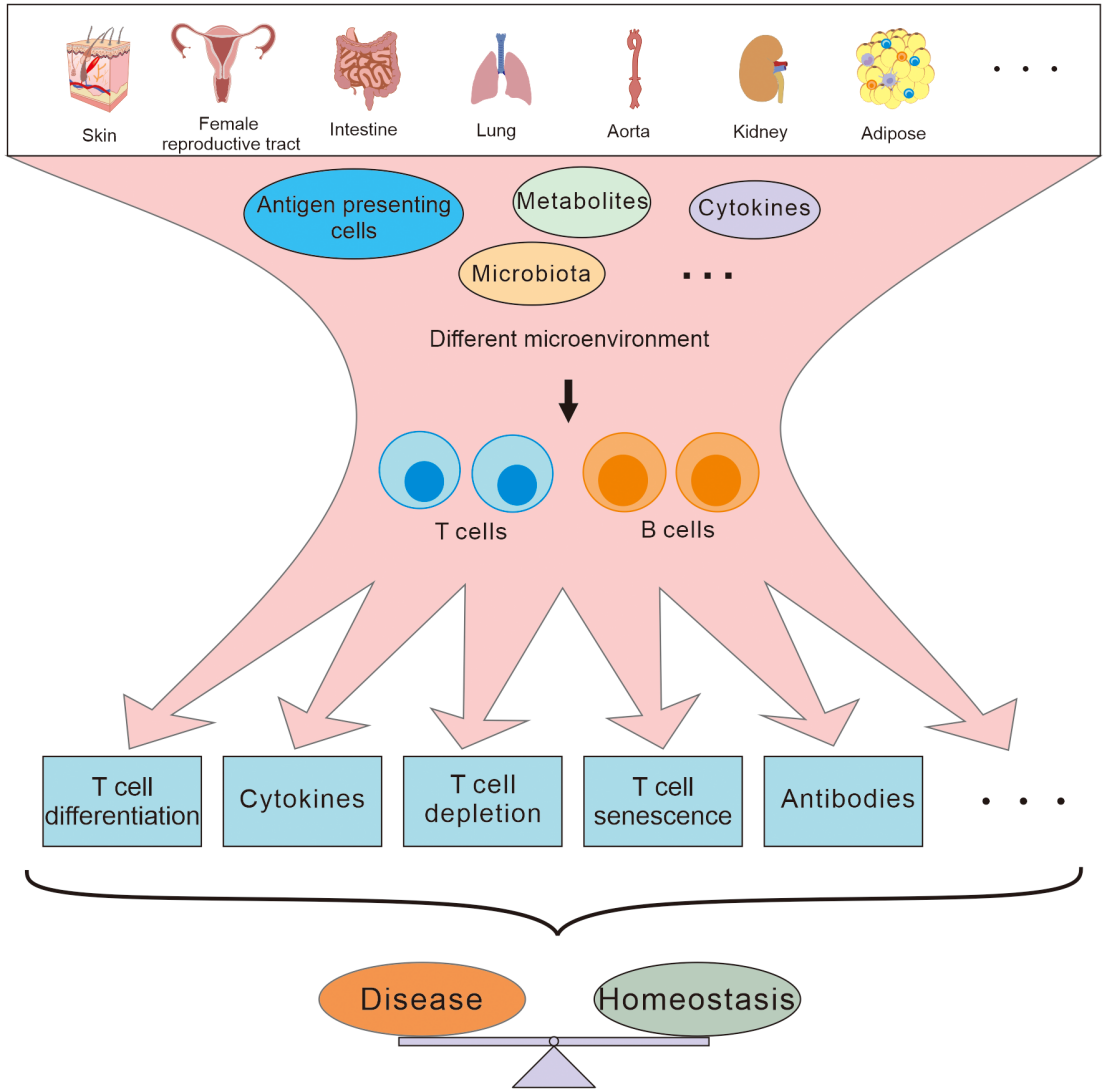
T and B cells residing in different peripheral tissues are involved in maintaining the homeostasis and disease development in different ways depending on the different microenvironment.

Several reviews and original research articles on adaptive immunity in various peripheral tissues are presented in this Research Topic. These cover the phenotype and function of T or B cells in peripheral tissues, as well as their roles in disease and possible therapeutic approaches.

T cells play an important role in maintaining homeostasis and inflammatory responses as helper or effector cells in adaptive immunity, which are activated and regulated by antigen-presenting cells (APCs) via cognate antigens presented and cytokines secreted respectively. Depending on the different microenvironment in the peripherial tissues, T cells can differentiate into different subtypes with different functions such as promoting inflammation, mediating immune tolerance, supporting cellular immunity, or favoring humoral immunity (4; Sun et al.; Sun et al.).

Barrier tissues are high-risk sites for infection and inflammation as the first line of defense against pathogens, Neuwirth et al. reviewed the commonalities and differences of the interactions between APCs and T cells in a variety of barrier tissues, including the skin, intestine, and female reproductive tract, under both homeostatic and infectious conditions. They pointed out that there are significant differences between T cells and the subsets of dendritic cells in different barrier tissues, controlling the balance between immune tolerance and immune responses mediated by regulatory T cells and other effector T cells respectively. Shirakawa and Sano summarized the transformation of CD4^+^T cells in visceral adipose tissue as a result of obesity. It was shown that the functionalities of CD4^+^T cells were closely associated with endocrine and metabolic homeostasis or dysfunction in visceral adipose tissue as well as obesity-associated chronic inflammation. Obesity-associated microenvironmental conditions could result in CD4^+^ T cell depletion and accelerate CD4^+^T cell senescence. Understanding these unique changes of CD4^+^T cells in specific adipose tissues will assist in the development of drugs for obesity-related diseases.

Murayama et al. found that CD4^+^CD8^+^ Tfh cells, as a heterogeneous subpopulation of Tfh cells, were enriched in IgG4-related disease lesions of palatine tonsils. These CD4^+^CD8^+^ Tfh cells might regulate IgG4 production by memory B cells through cytotoxic effects and are potential targets for regulating regional humoral immunity.

Ma´rquez-Sa´nchez et al. summed up the functions of various adaptive immune cells, together with their innate counterparts, in abdominal aortic aneurysms, and concluded that there are special roles for angiotensin II and microbiota in the activation of adaptive immune responses in the vasculature and perivascular adipose, as well as in the regulation of this disease.

The results of Wang et al. led to the proposal that CARDS toxin activates a positive feedback loop of type I immune responses in the lung during M. pneumonia infection. This putative mechanism could be useful in future approaches to investigate immune interventions for M. pneumoniae pneumonia.

Liu et al. summarized recent advances in the insights on the role of T cells and their products in type 2 diabetic kidney disease, pointing out that T cells played protective or pathogenic roles through various means such as inducing insulin resistance, mediating podocyte injury, participating in fibrosis and regulating proteinuria, and T cell- directed therapies in type 2 diabetic kidney disease were established with preliminary results.

In addition to αβ T cells, which are restricted by MHC-II, T cells that play innate immune roles, such as γδ T cells and natural killer T cells, are prevalent in peripheral tissues and have been found to have important roles in regulating adaptive immunity (2, 5, 6). Chen et al. summarized the role of γδ T cells present in the mucosa and skin in a wide variety of vector-borne diseases. The paper pointed out that γδ T cells could secrete multiple cytokines for immune regulation, formed immune memory and responded rapidly by proliferation in secondary infections.

Understanding the characteristics of T cell responses in different tissues may be useful in identifying new therapeutic targets for translational gains. In this regard, studies and the development of drugs targeting T cells in periphery tissues may be of great interest. In a systematic review of the TCR-like antibodies and their application in identifying autoantigen-presenting APCs, Li et al. suggested that TCR-like antibodies could play an important role in the study and treatment of autoimmune diseases. Along the same line, Su et al. found that the regulation of Th2/Th22 differentiation by the Galectin-9/T cell immunoglobulin mucin-3 pathway in skin was closely associated with the development of atopic dermatitis. Furthermore, Yan et al. summarized the effects of platelets on various immune cells and suggest that platelets could regulate the production of leukocyte cytokines, depending on the severity of the disease.

In addition to T cells, tissue-resident B cells, the other arm of adaptive immunity, are also mentioned in this Research Topic. Lee and Oh reviewed the history, localization, origin, and markers of tissue-resident memory B cells, and summarized the unique characteristics of humoral immunity in peripheral tissues like skin, intestine, and female reproductive tract.

Although the important roles of regional adaptive immunity in disease have been recognized, there is still much to learn about the regulation of diseases by the regional adaptive immunity. This Research Topic makes timely selection of articles highlighting the current understanding of adaptive immunity in such disease-affected tissues as barrier tissue, visceral adipose tissue, and the vascular walls, and discusses possible research and therapeutic tools in these areas so that the better understanding of regional immunopathophysiology in the diseased tissues can be achieved for effective therapeutic intervention.

## Author contributions

All authors listed have made a substantial, direct, and intellectual contribution to the work and approved it for publication.
